# Introduction

**Published:** 1855

**Authors:** 


### JOURNAL OF PUBLIC HEALTH.

### INTRODUCTION.

1. The Journal of Public Health is issued under the earnest conviction that it is a work of which the English nation has much need. During the past ten or fifteen years a new fact has opened on the English mind. *The national wealth is in a great degree dependent on the national health.* This fact, which some less civilized nations have been acquainted with for many long ages past, but which has so lately been rightly understood and valued here, is already accomplishing results, which cannot in the end fail to add greatly to the happiness of our homes, to the health of all classes of the community, and to our prosperity as a great people.

2. Fortunately, also, the simpler and more solid views, which are now held by the more important sections of the people, are well calculated to encourage a sound and lasting reform in all matters connected with the health and well-being of mankind. Pestilences, which, according to the remnants of an old mythology, were the invincible and corrective agents of an angered Deity, are now seen to be the mere effects of human ignorance, conquerable and even extinguishable under the influence of an improved and extended knowledge of natural causes and effects. Legislative proceedings, which were once made up only of parchment documents, diplomatic learning, and an historical knowledge of parliamentary forms, are at length becoming tinctured with a notion that science is a reality, and has a world-wide mission; and that in the soldier's barrack, the sailor's berth, the artizan's dwelling, the noble's mansion, the city, and the town, certain improvements are wanting which no one can appreciate who has not an acquaintance with those physical and universal laws upon which the life and existence of all animated nature depend. Even the theory and practice of the Esculapian art, that first and fairest of all human occupations, are passing through a remarkable and important revolution. The tendency of medicine, which, a century ago, was directed toward the division of diseases into many hundred forms, and the formation of the most elaborate and complex nosologies, is being in this day reversed; and the whole meaning of modern medical inquiry is to prove that disease is a unity, with a variety of phenomena, and that the causes of disease are reducible to a few elementary forms. This philosophical yearning after fixed principles as to the nature of disease is influencing the whole system of medicine. If the elements of diseased action are few and simple, the principles of prevention or cure are, it is thought, few and simple also. The materia medica is thus undergoing a thorough revision and curtailment. The viper no longer yields its body to the formation of a "specific" medicinal broth; the philosophic oil of bricks has become a droll myth, despite its magnificent name and former great reputation; a smell of the blooming dog-rose is now held to be as remedial a proceeding as the deepest draught of an infusion of its lifeless leaves; and these, and a thousand other *remedia admirabilia* which we might mention, are gone or are going into the catalogue of the things that have been. Meanwhile, a new Pharmacopœia comes in sight, which all can read. Its principles are preventive, its objects wide, and its elements—some seven only, and the world's general property—are no more than—*Pure air—Proper nourishment—A regulated temperature—Bodily exercise—Cleanliness—Mental education—Good morals.* Thus, in some of the most important sections of the community, there is, as we before said, a general improvement and simplification of knowledge on those great and vital questions, upon the correct solution of which so much of the world's happiness and progress rests.

3. But knowledge itself, however improved, is of but limited use, unless it is largely distributed, and, "like light communicated from one torch to another, is presently caught and copiously diffused." Nor can it ever be so diffused, except by the agency of the press it be dispensed in an accessible form, and be written in language so simple that he who runs may read.

4. To fulfil these indications is the main object of THE JOURNAL OF PUBLIC HEALTH. We wish to supply the reader with sound information on Health subjects, written in simple language, and published in such a form that all who are really anxious to possess it may obtain it without difficulty.

5. The list of Contributors' names which accompanies the Prospectus affords, we trust, a sufficient guarantee that the intentions of this Journal admit of a satisfactory realization. At the same time, we have in prospect far more assistance, and the promised co-operation of many able investigators of those natural and mechanical sciences with which we shall have specially to deal.

6. THE JOURNAL OF PUBLIC HEALTH will be an organ of free opinion and liberal sentiments; but encumbered by nothing approaching to personality, venality, unfair criticism, or angry disputations with other journals and publications. In our estimation all men of science, who deserve the name, and most if not all public writers mean, in the main, well. We hold also that individual writers are rarely fairly criticised or appreciated in their own time; that everything which is false either by intention or by accident is sure to die out of itself; and that whatever is good and truthful and honest will inevitably in its time receive a just recognition; in a sentence, "that the great soul of the world is just."

7. To a host of kind and enthusiastic supporters, who have come forward to aid and assist in this the most critical part of our labours, our deepest, warmest thanks are due. Be it our pleasing task ever to draw these friendly bonds closer, and to encourage the two great principles, without which no undertaking in which many minds are occupied can prosper—friendship without fear, and freedom without war.

